# Investigating the Utility of the BrainEye Smartphone Eye Tracking Application and Platform in Concussion Management

**DOI:** 10.1186/s40798-025-00819-8

**Published:** 2025-03-11

**Authors:** Meaghan Clough, Jade Bartholomew, Owen White, Joanne Fielding

**Affiliations:** Department of Neuroscience, Central Clinical School, Monash University, Melbourne, VIC 3004 Australia

**Keywords:** Sports related concussion, Eye tracking, Smartphone-based eye tracking, Post-concussion management

## Abstract

**Background:**

Concussion is a common consequence of engaging in collision sports, with the often mild, transient nature of symptoms posing a considerable diagnostic and management challenge. This challenge is vastly magnified for athletes competing at grassroots/non-professional levels, who lack field side access to medical expertise in the assessment of a player’s capacity to continue playing or need for further medical attention. The aim of this pilot study was to evaluate the utility of the BrainEye application and hardware (BrainEye platform) as a concussion screening tool, specifically determining (1) its sensitivity and specificity with respect to identifying an individual with a clinically diagnosed concussion, (2) the stability of the platform through test completion/failure rates, and (3) its usability through operator feedback and uptake/integration into concussion management protocols.

**Results:**

Using the BrainEye platform, 348 male professional Australian Rules footballers from 10 Australian Football League (AFL) clubs completed 4 simple ocular protocols (pupillary light reflex, PLR; smooth pursuit eye movements, SMP; near-point convergence, NPC; horizontal gaze nystagmus, HGN) at baseline, prior to the onset of the 2022 AFL season, and following the clinical diagnosis of concussion throughout the season during a game/training/practice (*n* = 11 players immediately following a concussive event, and on 14 occasions 2–7 days following a concussive event). Although club participation and protocol adherence rates were suboptimal, with clubs citing COVID-19 restrictions and cumbersome hardware set-up as primary reasons for non-participation/missing data, a BrainEye score that derived from an algorithm combining smooth pursuit and pupillary light reflex measures, achieved 100% sensitivity relative to clinical judgement, in identifying all instances of clinically diagnosed concussion, and 85% specificity.

**Conclusions:**

Collectively, the results of this study suggest that by removing the requirement for add-on hardware and providing a smartphone-only option with direct feedback on performance to the user, the BrainEye application may provide a useful screening tool for sport-related concussion.

## Key Points

Concussion remains a considerable diagnostic and management challenge, especially for the millions of individuals who engage in collision sports at grass roots levels.

Using a platform supported BrainEye app, this study demonstrated promising sensitivity, specificity, and stability in identifying concussed individuals throughout an AFL playing season.

A smartphone only option of this technology may ultimately provide a much-needed screening tool for sport-related concussion.

## Background

Concussion is a traumatically induced disturbance of brain function that occurs when external forces to either the head or body provoke rapid acceleration-deceleration of the brain [1, 2]. Not surprisingly, concussion is an injury frequently encountered in sporting environments [1]. The mild, transient nature of sports related concussion (SRC) symptoms pose a considerable challenge when attempting to determine whether or not a player is concussed and whether any consequential disturbance in neurobiological function has fully resolved. This challenge is vastly magnified for the majority of collision sport athletes who compete at grassroots or non-professional levels, who unlike professional athletes have no medical support teams or sophisticated technologies to augment a diagnosis of concussion and monitor recovery. This has created a degree of concern within the community about the safety of ongoing participation in collision sports, especially given the growing awareness of the potential consequences of experiencing multiple concussive injuries. Arguably, this concern may be addressed, at least in part in sporting environments, by providing additional means by which to identify a player who may be concussed and require medical intervention/management.

Already forming an important component of the clinical examination of a potentially concussed player, assessment of ocular and eye movement changes may provide a sensitive screen for concussion. The brain circuits involved in the processing of vision and control of ocular motility are highly distributed, extending to include a large proportion of the cerebral cortex and subcortex. Many of these circuits are in regions especially vulnerable to a concussive event including the frontal lobes [3], responsible for more volitional aspects of movement, and the brainstem [4], responsible for the final programming of movement, as well as housing autonomic control nuclei and pathways regulating pupillary function. A concussion invariably manifests in a reduction in our capacity to generate and execute appropriate ocular responses, and a subset of well-recognized concussive symptoms reflect this including dizziness, blurred vision, and trouble focusing [5]. Of the myriad of ocular changes that might signal a concussion, among the most salient are those that impact the integrity of versional eye movements and the pupillary light reflex.

Unfortunately, at present, assessment of ocular and eye movement changes can only be made by trained medical professionals. The aim of this pilot study therefore was to investigate the utility of a portable, easy to use ocular measurement tool, the BrainEye platform, as a concussion screen for all players. The BrainEye platform incorporates a smartphone application that presents simple ocular protocols (pupillary light reflex, PLR; smooth pursuit eye movements, SMP; near-point convergence, NPC; horizontal gaze nystagmus, HGN), and assesses ocular function using artificial intelligence (AI)/machine learning technologies integrated within a cloud-based system. Notably, within the sports and fitness industry, a range of wearables and portable systems already use AI technologies to garner information on other bodily functions, including heart rate monitors, blood oxygen sensors, electrocardiogram monitors, and other types of sensors capable of signalling potential health hazards [6].

The utility of the BrainEye platform in the provision of a SRC screen was investigated by determining (1) its sensitivity and specificity with respect to identifying an individual with a concussion, relative to the current gold standard clinical diagnosis by a medical professional, (2) the stability of the BrainEye platform through test completion/failure rates, and (3) the usability of the BrainEye platform through operator feedback and uptake into concussion management protocols. By leveraging the ubiquitous accessibility of smartphones as well as their cost-effectiveness it may ultimately be possible to place a user friendly means of helping to identify a concussed individual in anybody’s hand - this is potentially game changing.

## Methods

### Design

**Fig. 1:**
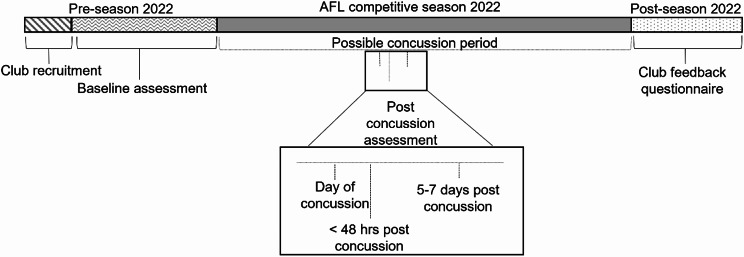
Study design and assessment protocol. Note, the timing of post-concussion assessment is indicative only. The actual timing of post-concussion assessment within the AFL 2022 season was dependent upon the occurrence of a concussion

This study was a prospective, longitudinal study conducted in male professional Australian Rules football players. Baseline assessments of all enrolled players were conducted by study researchers prior to the commencement of the 2022 Australian Football League (AFL) season (pre-season). Follow up assessments were conducted on players who sustained a clinically diagnosed concussion, as determined by their respective team doctor, during a game or training/practice within the 2022 AFL season. Follow up assessments were to be conducted by a team doctor or representative from the medical team immediately post-concussion (day of concussion), < 48 h post-concussion and 5–7 days post-concussion. No results were provided to club doctors or individual players. See Fig. 1 for an overview of the study design and assessment protocol. Designated club contacts received weekly reminder emails from study researchers to prompt compliance with the assessment protocol.

### Recruitment

Head doctors for all 18 AFL clubs were informed about the study by representatives of the AFL Concussion Innovation and Research team during the 2022 pre-season general medical team meeting. Study representatives subsequently contacted head doctors or nominated representatives via email with further details of the study requirements and commitments. Clubs and players were informed that club commitment did not extend to player commitment, with each player required to voluntarily opt-in to the study with signed informed consent. Study information packs were supplied to clubs for presentation to players prior to baseline assessments.

Ten clubs subsequently volunteered to be involved in the study. Eight clubs declined to be involved, 6 due to overstretched resources in the context of COVID-19 restrictions and added medical team responsibilities (75%), 1 due to a commitment to another concussion-based research program (12.5%), and 1 provided no response (12.5%). From the 10 participating AFL clubs, 348 professional male Australian Rules football players opted-in to the study. Individual players did not opt-in if they (1) were not an active player during the 2022 AFL season (2), were currently undergoing post-concussion protocols, or exhibiting post-concussion symptoms based on medical examination at the time of pre-season baseline assessment, or (3) for personal reasons. Each recruited player gave their informed consent prior to their baseline assessment, with all ethical procedures compliant with the ethical standards of the Monash University Human Research Ethics Committee (#29197) and the Helsinki Declaration of 1964 and its later amendments.

### BrainEye Platform

The BrainEye platform was developed to support the realisation of a smartphone only application. It comprised a BrainEye application installed on a data-enabled, android smartphone and the BrainEye hardware kit (see below). All clubs were provided with a Samsung, S21 android smartphone for the duration of the study.

#### BrainEye Hardware kit and Set-up

The BrainEye hardware kit is a simple to assemble, collapsible set-up comprising (1) a light bar that displays a series of LED lights for visual tracking (2), a chin rest and stand to ensure head stability and a uniform distance of 35 cm between participant and smartphone, and (3) a cradle that connects the smartphone to an inbuilt Infrared (IR)-enabled camera with flash. The hardware kit folds down for storage in a sturdy, compact case that conforms with airline carry-on guidelines. Additional requirements for assessment are (1) a table on which to mount the BrainEye platform (2), a height adjustable chair (3), access to a power source, and (4) access to WIFI or mobile data coverage. A distance of 35 cm between the chin rest and stand was chosen to maximise physical comfort and minimise convergence of the eyes while viewing on-screen stimuli.

**Fig. 2:**
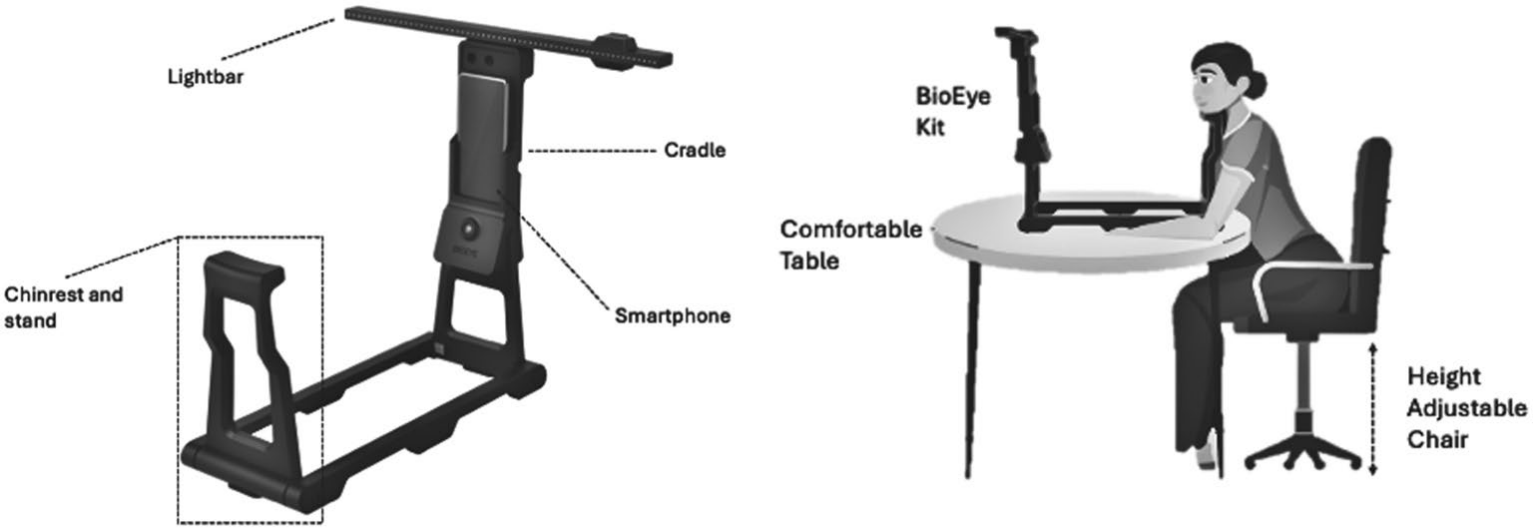
BrainEye hardware kit and assessment set-up. (**A**) BrainEye hardware kit along with all testing apparatus. The figure depicts the set-up for the Horizontal Gaze Nystagmus test. (**B**) BrainEye hardware kit assessment set-up

#### BrainEye Application

The BrainEye application is a cloud-connected smartphone application that collects ocular markers using an add-on IR enabled camera, with a high signal to noise ratio and excellent precision relative to scientific IR eye-trackers (r^2^ = 0.9-0.95). Using a bespoke AI/machine learning algorithm, the BrainEye application captures the spatial coordinates of the eye and pupil in real-time and translates these coordinates into meaningful ocular metrics. Specifically, it captures discrete pupil, iris, eyelid, and facial landmarks that, along with test-based target location information, are extracted and processed within cloud-based computing systems. User and environmental factors, such as head movement and lighting conditions, are measured and integrated within the cloud-based environment to account for individual differences and their impact on ocular metrics.

In this study, the BrainEye application delivered four discrete tests of ocular function, all of which are known to be affected post-concussion [7–14]: (1) Smooth Pursuit eye movements (SMP) (2), the Pupillary Light Reflex (PLR) (3), Near Point Convergence (NPC), and (4) Horizonal Gaze Nystagmus (HGN). Key measures for each test are outlined in Table 1.

1. SMP: continuous eye movements that smoothly track a moving target. This test was presented directly on the smartphone screen, with gaze maintained on a red target (dot) moving in a circle at 150 deg/sec (this speed approximates that used by Stubbs et al. and Maruta et al. which use a similar circular stimulus [15, 16]. Over the course of 1 min there were 7 sudden and randomly timed changes in target direction.
2. PLR: constriction of both pupils in response to the onset of a light stimulus, followed by a refractory dilation period after stimuli offset. A 3 sec light stimulus (flash) was provided by the cradle with gaze maintained on an on-screen target prior to, during, and following the flash. Total test time was 18 sec.
3. NPC: conjugate convergence of the eyes as they track a target moving closer to the face. A moving target was provided by the add-on light bar attached to the top of the cradle (at 90 degrees to the face plane). Here gaze was fixated on a series of LEDs that were illuminated serially, moving towards and away from the face. Total test time was 1 min.
4. HGN: biphasic ocular oscillations triggered by holding gaze peripherally. The target was provided by the add-on light bar attached to the top of the cradle (in the same plane as the face– see Fig. 2), with a series of LEDs illuminated sequentially for 1 sec from left to right and back, requiring a shift of gaze to each LED. At each end of the light bar the furthest LED was illuminated for 3 sec holding with gaze peripherally for 3 sec each. Total test time was 1 min.

**Table 1:** Description of all BrainEye application test measures

Measure	Description
Scores	
PLR score (%)	Derived from all individual PLR measures. Presented as a percentage with lower scores corresponding with poorer performance
SMP score (%)	Derived from all individual SMP measures. Presented as a percentage with lower scores corresponding with poorer performance
NPC score (%)	Derived from all individual NPC measures. Presented as a percentage with lower scores corresponding to poorer performance
HGN score (Y/N)	Y indicates presence of nystagmus. N indicates absence of nystagmus
BrainEye score (%)	Derived from SMP and PLR measures. Presented as a percentage with lower scores corresponding with poorer performance
**Pupillary** ** Light Reflex (PLR)**	
Latency (ms)	Time taken to initiate pupil constriction
Time to max constriction (ms)	Time taken to reach maximum pupil constriction
Total constriction (baseline (mm): peak constriction (mm))	Overall extent of change in pupil size from onset of flash until peak constriction
Baseline pupil size left/right (mm)	Size of left/right pupil before the flash (baseline)
Total redilation (peak constriction (mm): 3 s redilation (mm))	Overall extent of change in pupil size from peak constriction to maximal diameter within the subsequent three seconds
Redilation velocity (mm/ms)	Average velocity of pupil redilation from peak constriction to maximal diameter within the subsequent three seconds
**Smooth Pursuit (SMP)**	
Left/right correlation (Pearson’s r)	Correlation between location of eye and target– left/right eye
Pursuit velocity left/right (rad/ms)	Angular velocity of the eye divided by the angular velocity of the target– left/right eye
Pursuit lag left/right	Absolute difference between the eye and dot angle– left/right eye
Latency left/right (ms)	Average time taken for the eyes to correctly change direction when the target suddenly changes direction– left/right eye
Latency variation left/right (ms)	Average variation in time taken for the eyes to correctly change direction when the target suddenly changes direction– left/right eye
Pursuit error left/right	Euclidean distance between the eye and target– left/right eye
**Near Point Convergence (NPC)**	
Regain of convergence (cm)	Distance of LED target to face where convergence of the eyes was regained
Loss of convergence (cm)	Distance of LED target to face where convergence of the eyes was lost
**Horizontal Gaze Nystagmus (HGN)**	
**Presence of Nystagmus (Y/N)**	Y indicates presence of nystagmus. N indicates absence of nystagmus

Abbreviations: PLR: Pupillary Light Reflex; SMP: Smooth Pursuit eye movements; NPC: Near Point Convergence; HGN: Horizontal Gaze Nystagmus; ms: milliseconds; mm: millimetres; cm: centimetres

#### Feedback Questionnaire

A feedback questionnaire completed by clubs determined whether or not a club conducted the BrainEye tests throughout the 2022 AFL season, and if so, what that experience was like. If not, it determined the reasons why there was limited uptake. Clubs were also asked whether they would be more likely to use the BrainEye application if it was a phone-only device (i.e., no hardware required) and delivered immediate feedback on the players’ performance.

### Statistical Analyses

All data were generated by the BrainEye cloud-based analysis platform according to the methods described above. Table 1 outlines all measures generated for each test. IBM SPSS statistics package version 24 was used for all statistical analyses. Descriptive statistics (mean, standard deviation and 95% confidence intervals) were calculated for all test measures at all assessment time points.

The diagnostic utility of the BrainEye application was determined by calculating sensitivity, specificity, accuracy, and positive/negative predictive values, relative to the current gold standard clinical diagnosis by a medical professional. Receiver operating characteristics (ROC) statistics were used to determine the diagnostic capacity and cut-off scores for each derived score (PLR score, SMP score, BrainEye concussion score) as well as individual test measures (under the null hypothesis of area under the curve (AUC) of 0.5). Optimal cut-offs for each derived global score were selected by the highest Youden *J i*ndex value: (sensitivity + specificity − 1)*100. Sensitivity, specificity, positive predictive value (PPV), negative predictive value (NPV) were calculated as follows:

$$\:Sensitivity:\:\left(\frac{TP}{TP+FN}\right)*100\:Specificity:\:\left(\frac{TN}{TN+FP}\right)*100$$

TP: true positive TN: True negative.

FP: false positive FN: False negative

$$\eqalign{& {\rm{Positive }}\,{\rm{predictive }}\,{\rm{value }}\left( {{\rm{PPV}}} \right){\rm{:}} \cr & \left( {{{{\rm{Sensitivity*prevalence}}} \over {\left( {{\rm{Sensitivity*prevalence}}} \right){\rm{ + }}\left( {\left( {{\rm{1 - specificity}}} \right){\rm{*}}\left( {{\rm{1 - prevalence}}} \right)} \right)}}} \right) \cr} $$

$$\eqalign{& {\rm{Negative}}\,{\rm{predictive}}\,{\rm{ value}}\,{\rm{ }}\left( {{\rm{NPV}}} \right){\rm{:}} \cr & \left( {{{\left( {{\rm{Specificity*}}\left( {{\rm{1 - prevalence}}} \right)} \right)} \over {\left( {\left( {{\rm{1 - sensitivity}}} \right){\rm{*prevalence}}} \right){\rm{ + }}\left( {{\rm{specificity*}}\left( {{\rm{1 - prevalence}}} \right)} \right)}}} \right) \cr} $$

$${\rm{Accuracy:}}\left( {{{{\rm{TP + TN}}} \over {{\rm{TP + TN + FP + FN}}}}} \right)$$

*Prevalence was determined as the proportion of concussed players.

The utility of the BrainEye application and associated hardware was also assessed through test completion/failure rate. Successful completion was defined as the return of a result indicating accurate AI/machine learning eye tracking analysis. Completion failure was defined as the return of a null result indicating; [1] failure of AI/machine learning eye tracking analysis due to participant factors (e.g., moving, blinking etc.) or software failure [2], failure of BrainEye kit hardware. Finally, utility of the application was assessed in terms of [1] use by clubs throughout the season (protocol uptake into post-concussion management, completion rates), and [2] feedback provided by clubs post season.

Finally, change in baseline and post-concussion test performance over time was assessed using non-parametric Wilcoxon signed-rank test and reported with Cohen’s d effect sizes.

## Results

**Fig. 3:**
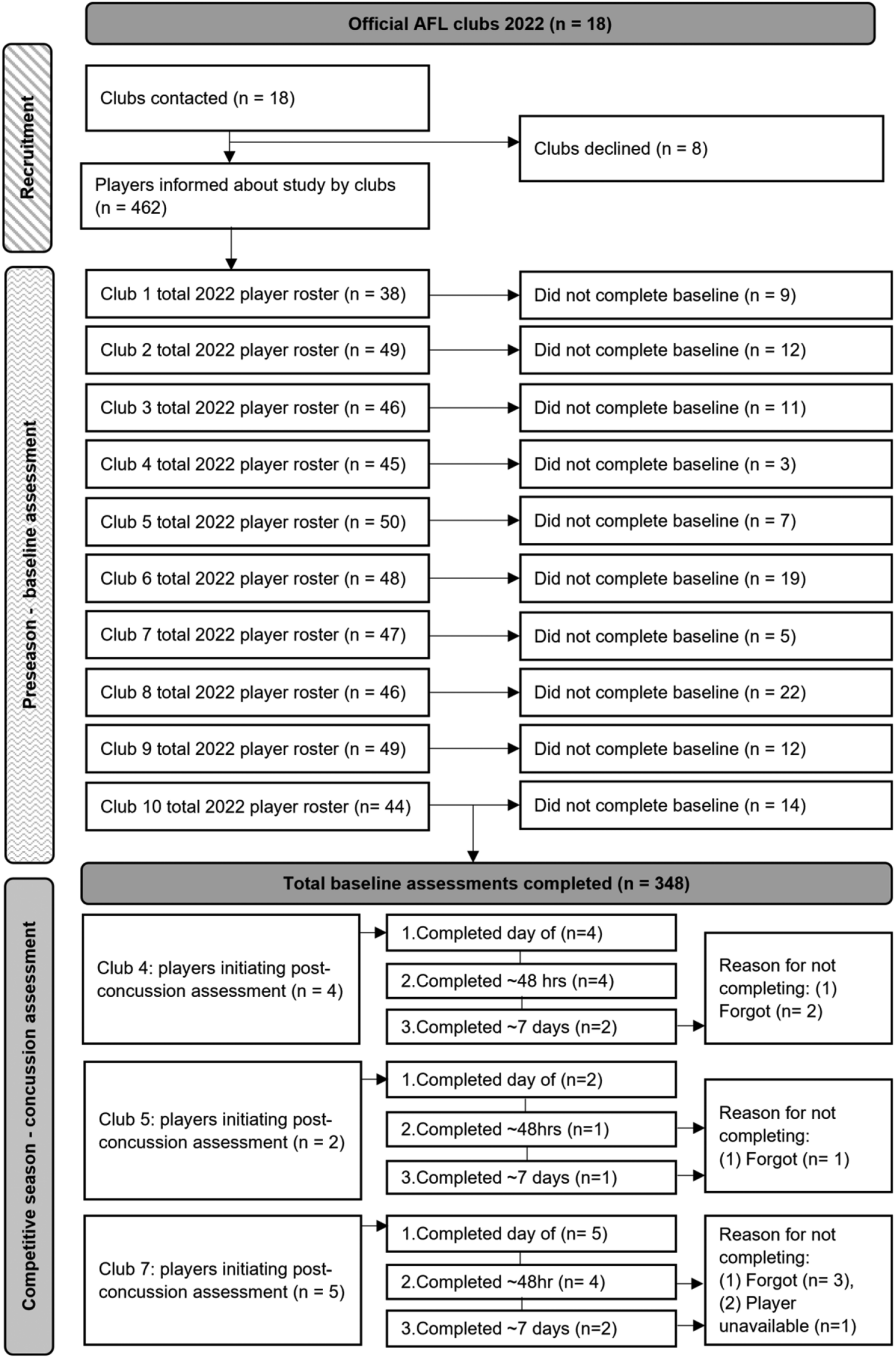
Study recruitment and protocol adherence

A full breakdown of study recruitment and protocol completion at both baseline and post-concussion can be found in Fig. 3.

From the 10 clubs that volunteered to participate, a total of 462 players were registered on 2022 season club rosters. Of these, 384 (83%) opted-in to the study and completed baseline assessments. The distribution of players recruited across clubs was relatively even, with more than 50% of eligible players completing baseline assessment for all clubs; for 7 clubs, 75–95% of their players were assessed at baseline (clubs #: 1,2,3,4,5,7,9), and 3 clubs had 52–68% of players assessed at baseline (clubs #: 6,8,10).

### Baseline Completion and Player Performance

All baseline data can be found in Table 2.

#### BrainEye Application Baseline Completion

Players performing both the SMP and PLR tests achieved high completion *success* at baseline, with approximately 90% of players returning useable results. For the PLR, the primary reasons for unusable data were failure to detect onset of constriction due to blinking, and/or failure to detect pupil due to hair/eyelashes obscuration (total *n* = 37). For SMP, the primary reasons for unusable data were excessive head movement and/or issues with the BrainEye landmark detection model (total *n* = 32).

In contrast, for both NPC and HGN, high completion *failure* and unusable data were found at baseline, with successful completion only achieved by 19% and 47% of players respectively. For NPC, the primary reasons for unusable data were players not adhering to test instructions, eye closure, looking away from target during the test (*n* = 102), or issues with the BrainEye hardware kit (*n* = 181). For HGN, the primary reason for unusable data was issues with the BrainEye hardware kit (*n* = 187). Due to the high likelihood of missing data bias, no further analyses of the NPC and NPS tests were conducted.

#### Baseline Player Performance

Player performance on the SMP and PLR tests was not significantly different across clubs for any test measure or overall score. As such, all baseline data were combined for further analyses.

**Table 2:** Player demographic and baseline BrainEye EyeCon application measures

	M	SD
Age	24.95	1.08
**Pupillary** ** light reflex**	**M**	**SD**
Latency(ms)	410.10	135.06
Max constriction time(ms)	1553.50	609.5
Total constriction	0.94	0.42
Right pupil size	4.36	0.70
Left pupil size	4.34	0.68
Total redilation	0.64	0.31
Redilation velocity	0.25	0.11
**Smooth pursuit**	**M**	**SD**
Left correlation	0.78	0.07
Right correlation	0.77	0.09
Pursuit velocity right	1.45	0.29
Pursuit velocity left	1.51	0.35
Pursuit lag right	0.62	0.21
Pursuit lag left	0.62	0.19
Latency direction change right	364.06	48.84
Latency direction change left	371.03	46.10805
Latency direction change variation right	56.21	18.42
Latency direction change variation left	56.30	18.10
Pursuit error left	0.89	0.20
Pursuit error right	0.88	0.18
**Near Point Convergence**	**M**	**SD**
Loss of convergence	4.06	4.10
Regain of convergence	5.18	5.34
**Horizontal Gaze Nystagmus**	**Yes**	**No**
Nystagmus presence (Yes/No)	-	100%
**Aggregate test scores**	**M**	**SD**
PLR score (%)	93.87	5.08
SMP score (%)	93.45	10.09
BrainEye Concussion Score (%)	94.7	12.28

## Discussion

This study aimed to investigate the utility of the BrainEye platform as a screening measure for SRC. We demonstrated that an overall measure of function, the BrainEye score, derived from smooth pursuit (SMP) and the pupillary light reflex (PLR) measures, accords with clinical judgement, identifying all concussed player data sets (100% sensitivity). Relatively lower specificity (84.6%) indicates that the BrainEye score is, at present, overly inclusive, with an approximately 15% false positive rate. While any false positive rate is problematic for a clinical diagnostic test, in the context of a screening measure, it is immeasurably preferable to have lower specificity, with the consequence of a player misclassified as not concussed far outweighing those of being misclassified as potentially concussed. In short, it is better to over diagnose concussion rather than under diagnose concussion. The worst-case scenario is that a non-concussed player might leave the field, seek medical attention, and be subsequently cleared by a medical professional.

It is imperative at this point that the limitations of study are acknowledged. Although these should not detract from the robustness of our findings, club participation and incorporation of the BrainEye application into post-concussion management protocols was significantly impacted by the necessity to also adhere to COVID-19 protocols. Specifically, participation was compromised by the already demanding additional requirements of medical teams in a COVID-19 impacted space. Accordingly, we received relatively few concussion datasets and performed post-concussion analyses against baseline data rather than matched comparison data (i.e., non-concussed individuals at the time of testing). Further, club doctors were not blind to diagnosis.

However, ocular and eye movement changes are widely recognised following concussion, thought to underlie some of the more salient changes like dizziness, blurred vision and impaired coordination. These changes reflect disruption to a widely distributed neural network, incorporating many regions especially vulnerable to a concussive event. SMP eye movements, for example, implicate long ranging connections spanning the visual pathways from retina to cortex, brainstem, cerebellum, basal ganglia and multiple visual, temporal, parietal and frontal cortical regions. Given the complexity of the system, it is perhaps unsurprising that a range of SMP metrics distinguish concussed from non-concussed individuals. Although previous studies have presented several different types of stimuli (circular horizontal, vertical, sinusoidal, step ramp), many different velocities and stimuli sizes/shapes, results variously demonstrate that following a concussion, SMP eye movements may be inaccurate (9, 13, 10, 16–18), be dysconjugate (eyes movements are asymmetrical) [16, 19, 20], have increased initiation latencies [18], and exhibit greater intra-individual variability in accuracy [18].

Pupil abnormalities are also common immediately following a concussion and while they often resolve over a short period time, can persist indefinitely. Notably, pupillary size, shape, and reactivity to light are regulated by the autonomic nervous system - central autonomic control nuclei and pathways are mainly integrated within the brainstem, shown in histopathological studies to be an important site of axonal injury following concussion. Although studies have tested subjects with different stimulus conditions, at various ages, and at various timepoints following concussion, results following a concussion variously demonstrate changes to the following: pupillary changes to steady-state pupil size [21–23], pupil size following constriction in response to a light stimulus [21–23], time to maximum constriction in response to a light stimulus [23], peak and average constriction velocities [21–25], peak and average dilation velocities [21, 23, 24], and constriction amplitudes [21].

The increasing sophistication of AI technologies make it possible to monitor basic human functions with ease and accuracy. Professionally, the added value of introducing an objective measure of function like the BrainEye application, once validated, into the arsenal of tools available to enhance diagnostic certainty is invaluable. Clinical evaluation, the current gold standard measure of concussion, is at present widely complemented by performance on the Sports Concussion Assessment Tool (SCAT). However, despite being widely accepted and commonly used, the suitability of the SCAT is questionable. This can be attributed to several issues including practice effects when conducted serially over a short period of time, insensitivity to subtle change in performance, subjectivity and unreliability, and the possibility of a player purposely modifying pre-season/match performance to evade a positive diagnosis, and missing play [25–27]. At the grassroots or non-professional level, in the absence of a health professional, any indication of reduced function following a head knock is potentially lifesaving.

Of course, the limitations of not only the study environment but the application of the technology itself must be acknowledged. Firstly, the BrainEye application was supported by an infrared add-on and replication of these results is required with the smartphone camera only. Further, the BrainEye application is not intended as a diagnostic tool. Diagnosis of concussion remains a clinical decision. Finally, this study sample was limited in terms of representing the wider community, and the sample size small. The prevalence of concussion is also poorly reflected here.

## Conclusion

Despite the aforementioned limitations, the finding of perfect sensitivity (100%) and accuracy (90%) relative to gold standard clinical judgement highlights the power of the BrainEye smartphone-based eye tracking technology as a method for screening concussions in the absence of expert medical personnel. This has particular significance for grassroots level, non-professional athletes who when faced with a suspected concussion lack expert medical input to make important health decisions. Finally, the important insights from the medical teams in this study highlight the need for further development of the BrainEye application, transitioning away from the need for the add-on platform to a smartphone only application. Such an application could provide valuable diagnostic information that is accessible irrespective of medical training.

### Post-Concussion Assessment

#### Protocol Integration into Post-Concussion Management and Completion

Of the 10 AFL clubs who volunteered to participate only 3 clubs integrated the protocol into their post-concussion management of players. Eleven players completed at least 1 post-concussion assessment. All 11 concussed players were assessed on the day of diagnosis (*n* = 11), 82% were assessed 48 h post-concussion (*n* = 9), and 45% were assessed 5–7 days post-concussion (*n* = 5). Reasons for not integrating the protocol into post-concussion management were that the club medical team representative forgot to conduct assessment (*n* = 6), or that the player was unavailable for follow up assessment (*n* = 1). [See Fig. 3].

#### Club Feedback

Of the 7 clubs who did not complete any post-concussion assessments, 5 clubs provided feedback regarding their lack of protocol integration into their post-concussion assessment. All clubs except 1 stated that they found the kit and set-up too difficult and/or time consuming to incorporate into their existing post-concussion assessment protocol. Of note, when asked what would improve uptake and integration of the BrainEye platforms into post-concussion protocols, all clubs including those who did complete post-concussion assessment, stated that a movement to a smartphone only application would be preferable.

### Diagnostic Sensitivity and Specificity of the BrainEye Application Measures and Scores for Concussion

Diagnostic accuracy, optimal cut-off values, sensitivity and specificity information can be found in Table 3.

**Fig. 4:**
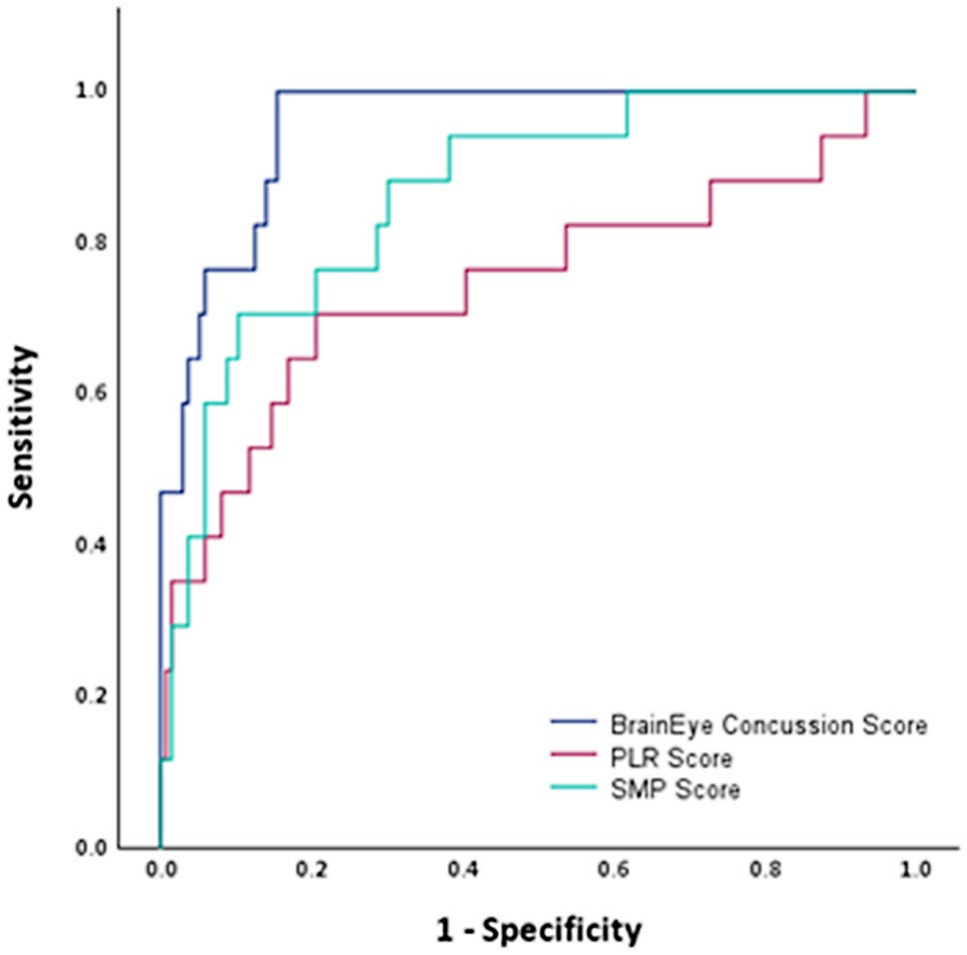
AUC for PLR, SMP and BrainEye concussion scores. Abbreviations: PLR: Pupillary Light Reflex; SMP: Smooth Pursuit eye movements

PLR, SMP and BrainEye concussion score demonstrated an AUC significantly greater than 0.5 indicating diagnostic accuracy significantly greater than chance for identifying players with concussion (see Fig. 4). Further, these scores achieved a Youden *J* index of 50% or above meeting the empirical benchmark for diagnostic purposes, with the BrainEye concussion score achieving the highest value (84.56%). Individually, the PLR and SMP scores produced moderate sensitivity and specificity (70% and 79–89% respectively), as well as PPV, NPV and accuracy. In contrast the BrainEye concussion score had perfect sensitivity (100%), and high specificity (85%), accuracy (91%), PPV (86%) and NPV (100%).

Six out of the 12 individual SMP measures demonstrated diagnostic accuracy for concussion significantly greater than chance: left/right correlation, left lag, right/left latency, left error. Overall, the sensitivity of these measures was low (~ 55%), and the specificity moderate (~ 75%). [Figure 4].

Five out of the 7 individual PLR measures demonstrated diagnostic accuracy for concussion significantly greater than chance: max constriction time, total constriction, right/left pupil size, total redilation. On average the sensitivity of these individual measures was moderate to low (~ 60%), except for total redilation, which demonstrated a sensitivity of 95%. Similarly, specificity for all measures was a little higher (~ 70%), but still moderate.

**Table 3:** Area under the curve values, sensitivity, specificity and cut-off values for BrainEye test measures and overall scores immediately post-concussion

	AUC	S.E	95% CI	Youden J	Sensitivity	Specificity	PPV	NPV	Accuracy	Cut-off values
**Scores**										
PLR score	0.75**	**0.08**	**0.6-0.8**	**50.00**	**70.6**	**79.4**	**77.4**	**72.9**	**75**	**90.37**
SMP score	0.87**	**0.04**	**0.8-0.9**	**60.29**	**70.6**	**89.7**	**87.2**	**75.3**	**75.3**	**83.72**
BrainEye Concussion score	0.95**	**0.02**	**0.9 − 1**	**84.56**	**100**	**84.6**	**86.6**	**100**	**90.8**	**89.05**
**Pupillary** ** light reflex (PLR)**										
Latency (ms)	0.533	0.07	0.4-0.7	13.13	27.3	85.9	65.9	54.1	44.2	291.9
Max constriction time	0.71*	**0.07**	**0.6-0.8**	**41.00**	**72.7**	**68.3**	**69.6**	**71.4**	**69.3**	**1155.3**
Total constriction	0.64*	**0.06**	**0.5-0.7**	**26.21**	**50.0**	**76.2**	**67.7**	**60.3**	**67.7**	**0.6**
Right/Left pupil size	0.70*	**0.06**	**0.6-0.8**	**43.01**	**68.2**	**74.8**	**73.01**	**70.1**	**73.1**	**3.8**
Total redilation	0.66*	**0.05**	**0.6-0.7**	**36.83**	**95.5**	**41.4**	**61.9**	**90.1**	**47.9**	**0.68**
Redilation velocity	0.58	0.06	0.5-0.7	18.31	27.3	91.0	75.2	55.5	58.2	0.12
**Smooth pursuit (SMP)**										
Left correlation	0.75**	**0.05**	**0.7-0.8**	**41.8**	**83.3**	**58.5**	**66.7**	**77.7**	**63.0**	**0.38**
Right correlation	0.65*	**0.07**	**0.5-0.8**	**32.97**	**44.4**	**88.5**	**79.4**	**61.4**	**71.4**	**0.95**
Pursuit velocity right	0.45	0.08	0.4-0.6	14.74	26.3	88.4	69.3	54.5	56.3	-1.04
Pursuit velocity left	0.45	0.08	0.3-0.6	13.16	15.8	97.4	85.8	53.6	43.4	-1.18
Pursuit lag right	0.59	0.07	0.5-0.7	26.32	47.4	78.9	69.1	60	68	− 0.80
Pursuit lag left	0.67*	**0.06**	**0.6-0.8**	**30.53**	**57.9**	**72.6**	**67.8**	**63.2**	**68.4**	**− 0.62**
Latency direction change right	0.63	0.06	0.5-0.7	25.26	52.6	72.6	65.7	60.5	71.4	− 0.65
Latency direction change left	0.68*	**0.06**	**0.6-0.8**	**27.90**	**47.4**	**80.5**	**70.8**	**60.4**	**68.9**	**− 0.89**
Latency direction change variation right	0.65*	**0.07**	**0.5-0.7**	**31.42**	**50.0**	**81.4**	**72.8**	**61.9**	**70.7**	**0.63**
Latency direction change variation left	0.54	0.07	0.4-0.6	13.57	27.8	85.8	66.1	54.3	57.1	0.88
Pursuit error left	0.65*	**0.06**	**0.6-0.8**	**29.47**	**63.2**	**66.3**	**65.2**	**64.3**	**65.5**	**− 0.55**
Pursuit error right	0.58	0.07	0.5-0.7	21.05	52.6	68.4	62.4	59.06	63.8	− 0.52

^*p <.05, ** *p* <.001. AUC: Area Under the Curve, Cut−off scores for measures are optimal derived cut−off scores^

Abbreviations: S.E: Standard error; PPV: Positive predictive value; NPV: Negative predictive value; CI: Confidence interval

### Comparison Between Baseline and Post-Concussion BrainEye Platform Performance in Concussed Players

**Fig. 5:**
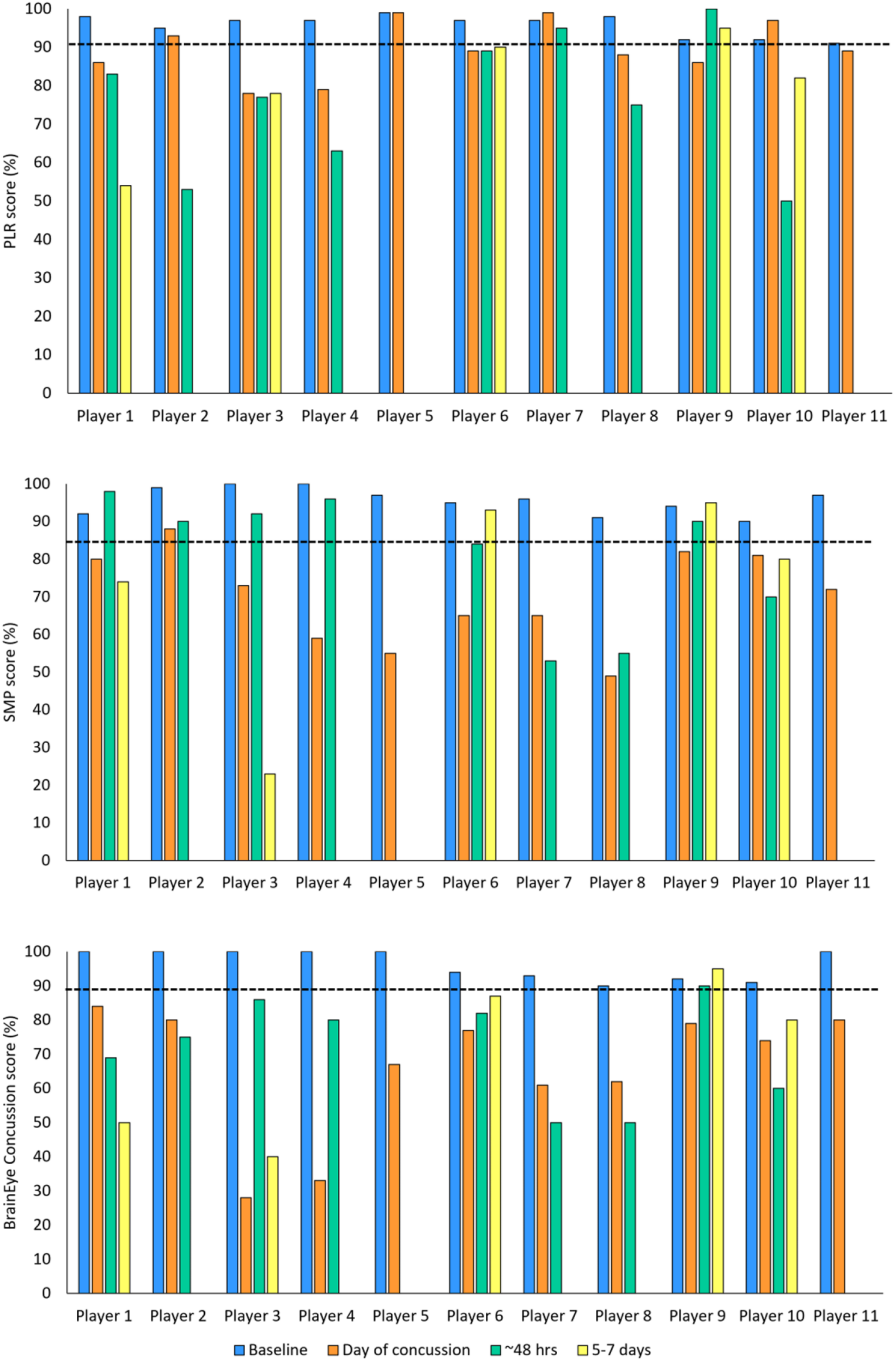
Graphical depiction of individual scores for each player at each time-point. Dotted lines indicate derived cut-off scores for each tests

At baseline, none of the 11 concussed players performed below cut-off for any score. Post-concussion, all players had a concussion score below cut-off on the day of concussion (100%), 9 at 48 h, and 5 at 5–7 days (see Fig. 5; Table 4).

For PLR score, a significant decrease in score was found between baseline and day of concussion (Z = -2.2, *p* =.03, Cohen’s d = 1.2; large effect), and baseline and ~ 48 h post-concussion (Z = -2.4, *p* =.02, Cohen’s d = 1.54; large effect). No significant difference was found between baseline and 5–7 days post-concussion (Z = -1.7, *p* =.08, Cohen’s d = 1.3; large effect).

Similarly, for SMP score, a significant decrease in score was found between baseline and day of concussion (Z = -2.9, *p* =.003, Cohen’s d = 2.8; large effect), and baseline and ~ 48 h post-concussion (Z = -2.3, *p* =.02, Cohen’s d = 1.17; large effect). No significant difference was found between baseline and 5–7 days post-concussion (Z = -1.7, *p* =.08, Cohen’s d = 1.1; large effect).

Finally, for BrainEye concussion score, a significant decrease in score was found between baseline and day of concussion (Z = -2.9, *p* =.003, Cohen’s d = 2.2; large effect), and baseline and ~ 48 h post-concussion (Z = -2.7, *p* =.008, Cohen’s d = 2.3; large effect). No significant difference was found between baseline and 5–7 days post-concussion (Z = -1.7, *p* =.08, Cohen’s d = 1.5; large effect).

**Table 4:** Baseline and post-concussion performance for concussed players (including overall averages and standard deviations)

Concussed player	PLR score (%)				SMP score (%)				BrainEye Concussion score (%)			
	Baseline	Day of concussion	~ 48 h	5–7 days	Baseline	Day of concussion	~ 48 h	5–7 days	Baseline	Day of concussion	~ 48 h	5–7 days
Player 1	98	86*	83*	54*	92	80*	98	74*	100	84*	69*	50*
Player 2	95	93	53*		99	88	90		100	80*	75*	
Player 3	97	78*	77*	78*	100	73*	92	23*	100	28*	86*	40*
Player 4	97	79*	63*		100	59*	96		100	33*	80*	
Player 5	99	99			97	55*			100	67*		
Player 6	97	89*	89*	90	95	65*	84	93	94	77*	82*	87*
Player 7	97	99	95		96	65*	53*		93	61*	50*	
Player 8	98	88*	75*		91	49*	55*		90	62*	50*	
Player 9	92	86*	100	95	94	82*	90	95	92	79*	90	95
Player 10	92	97	50*	82*	90	81*	70*	80*	91	74*	60*	80*
Player 11	91	89*			97	72*			100	80*		
Average	95.7	89.3	76.1	79.8	95.5	69.9	80.9	73	96.4	65.9	71.3	70.4
Standard deviation	2.8	7.2	17.8	15.9	3.5	12.4	17.3	29.3	4.3	19.1	15.04	24.05

^*score is below derived cut off^

Abbreviations: PLR: Pupillary Light Reflex; SMP: Smooth Pursuit

## Data Availability

The datasets generated and analysed during the current study are not publicly available due to contractual restrictions concerning privacy of participant medical information and proprietary information held by BrainEye.

## Abbreviations

SRC: Sports related concussion
PLR: Pupillary light reflex
SMP: Smooth pursuit eye movements
NPC: Near-point convergence
HGN: Horizontal gaze nystagmus
AI: Artificial intelligence
AFL: Australian Football League
IR: Infrared
AUC: Area under the curve
TP: True positive
TN: True negative
FP: False positive
FN: False negative
PPV: Positive predictive value
NPV: Negative predictive value
SCAT: Sports Concussion Assessment Tool
